# What if some patients are more “important” than others? A possible framework for Covid-19 and other emergency care situations

**DOI:** 10.1186/s12910-022-00763-2

**Published:** 2022-03-13

**Authors:** Andrea Lavazza, Mirko D. Garasic

**Affiliations:** 1 Centro Universitario Internazionale (CUI), Cui, via Garbasso, 42, 52100 Arezzo, Italy; 2 University of Pavia, Pavia, Italy; 3 Dipartimento di Scienze della Formazione, Roma Tre University, Rome, Italy

**Keywords:** Triage, Altruism, Healthcare renunciation, Priority treatment, Informed consent

## Abstract

**Background:**

The Covid-19 pandemic caused situations where, in some hospitals, there were more patients in need of urgent treatment in intensive care units (ICU) than were available. In particular, there were not sufficient ventilators or critical care resources for all patients in danger of dying from respiratory failure or other organ failures.

**Discussion:**

As the “first come, first served” criterion was not considered adequate, more nuanced and fairer clinical criteria were proposed to assess whom to treat first. One type of patients that has not been considered in the literature so far is that of “important patients”, individuals that many people might consider worthy of priority treatment for the contribution they made or might make to society as a whole.

**Summary:**

In this article, we discuss the moral insights behind the possible treatment of “important patients” and suggest a supererogatory solution of voluntary renunciation/withdrawal. Details of the proposal are explained, and potential objections are addressed.

## Background

The Covid-19 pandemic has brought to light some uncommon dilemmas that ethical reflection can now attempt to analyse. Many cases have already been considered with regards to the allocation of scarce resources. In particular, attention has been focused on the prioritisation of patients most likely to survive over those with remote chances [1–4]. Here we want to deal with a topic that has not been dealt with so far from the point of view of moral evaluation, although it has certainly been the subject of personal or implicit considerations [2, 5, 6].

The topic we want to address has to do with people whom we will tentatively call “important” and/or “famous”. The dilemma that “important or famous patients” may pose to medical or political decision-makers falls within a well-known scenario that has been the subject of extensive reflection in relation to the Covid-19 pandemic: we call this clinical situation the “insufficient ventilators for all” scenario [7], or insufficient critical care resources scenario.^1^ In fact, intensive care units (ICU) have often been unable to admit new patients. This was combined with the impossibility of moving patients to other hospitals, both because they too were operating at full capacity, and because of time pressures: Covid-19 often causes a rapid worsening of respiratory conditions that requires fast intervention with respiratory support (ventilator) to prevent the death of the patient.

The coordinates of this scenario have been the subject of numerous analyses. Health authorities, resuscitator societies and national bioethics committees in many countries have established criteria for access to intensive care in cases where patients outnumber the available life-saving devices. In general, these should be universalistic criteria that do not involve discrimination and are rationally acceptable both from a deontological point of view (the premise being that some principles, such as that by which human beings can never be treated as a means, can never be violated) and from a consequentialist point of view (which involves assessing the highest expected utility and allowing some sacrifices to be made to achieve it).

For instance, when the Covid-19 crisis in Italy worsened at the beginning of March 2020, the Italian Society of Anaesthesia, Analgesia, Resuscitation, and Intensive Care (SIAARTI) predicted an increase in cases of acute respiratory insufficiency (requiring hospitalization in the ICU) of such magnitude as to cause a strong imbalance between the population’s clinical needs and the effective availability of intensive resources. Faced with this scenario, it was believed that it might be necessary to adopt “criteria for access to intensive care”, not only in terms of strictly clinical appropriateness and proportionality of care but also in terms of distributive justice and proper allocation of limited healthcare resources.^2^

Given the scenario akin to “disaster medicine”, SIAARTI proposed some clinical ethics recommendations for the allocation of intensive care treatment in exceptional, resource-limited circumstances. These were “an extension of the principle of proportionality of care, allocation in a context of a serious shortage of healthcare resources”, and the “aim at guaranteeing intensive treatments to patients with greater chances of therapeutic success”. In general, triage protocols include several principles and criteria—spanning across short term prognosis, survival in the ICU, long term prognosis, and number of life years saved (life expectancy)—which cannot be all epitomized in the expression “chances of therapeutic success”.

The need for intensive care, it is said in SIAARTI’s guidelines, must be also integrated with other elements of “clinical suitability”, thus including the type and severity of the disease, the presence of comorbidities, the impairment of other organs and systems, and their reversibility. This does not necessarily mean having to follow a criterion like “first come, first served” for access to intensive care. It is implicit—as the document underlines—that the application of rationing criteria is justifiable only after all the actors involved have made all possible efforts to increase the availability of resources and after every possibility of transferring patients to centres with greater availability of resources has been exhausted.

These types of guidelines, where choices are left to experts in the field, may generate a heated debate. Triage protocols sometimes stir discord as they can contradict the basic principles of life-saving medicine. In a sense, those protocols are taken to be discriminatory by some, for instance against physically or mentally impaired people, while many think that their implementation helps maximize benefit in terms of lives spared. However, the criteria offered by SIAARTI, like similar ones proposed in other countries, are recognized as quite reasonable and supported by the specialist knowledge of experts, who are the most qualified to make these choices, although there is always room for dissent and difference of opinion [9–12].

Considering all that, what we want to address is a so far overlooked topic, namely that of the status of “important” and/or “famous” patients with respect to the “insufficient ventilators for all” scenario and the possible guidelines that medical or political decision-makers might consider. We will begin with some insights into the status of that type of patient and then move on to assess how well founded it is and how we might possibly respond to it. The conclusion will be twofold. Only from a purely consequentialist point of view [13], which, however, conflicts with other widespread moral intuitions, can it be legitimate in some cases to take a respirator off a patient to give it to an “important and/or famous” one. Nevertheless, a supererogatory ethical protocol involving the informed and unbiased choice on the part of individual patients can be considered admissible. Accordingly, a specific protocol and procedures can be devised.

## Main text

### Special patients?

At the outset, it must be specified what is meant by “important and/or famous” patient. They are those who hold relevant public positions or positions that have an influence on a significant number of people, e.g., heads of state or government, religious leaders, major businessmen and the like. Scientists, intellectuals, and artists can also fall into this category, along with famous people in general such as professional athletes or people who are very present on television or social media and are therefore the object of attention or imitation for many. “Important” people are also those who have particularly distinguished themselves in philanthropy or helping others, or who are clear examples of good living.^3^

In addition, the concept of notoriety cannot simply be linked to how many people know a given individual. Many people may have heard of a politician who spied on opponents, or a sportsman disqualified for doping. These people would not be deemed “famous” in the positive sense we consider here. The case of social media influencers or celebrities who only appear in the media is different. In this case, the question would not be about their contribution to society but whether many people love them, and how many people (if any) would be in some way harmed by the death of the influencer or the celebrity.

When assessing the contributions of important and/or famous people, one element to consider is also whether more weight should be given to past contributions or future contributions. In general, the qualification of importance/notoriety is derived from past contributions, but certainly the possibility of contributing to society in the future should be taken into account. In this sense, a very old individual who is already in poor health or an individual who for some reason has already declared that they do not wish to continue with their activities, or persons who have recently been responsible for highly reprehensible acts, might not be considered eligible, whatever their past contributions may have been. There are, of course, cases that are not easy to decide upon, such as retired sportspeople or dancers (one could say that they might provide excellent teaching to new generations in their field).

It seems like there are no unambiguous and quantifiable criteria to determine who is important and/or famous. That is why we propose to rely on an ad hoc committee selected through a very careful procedure (more on this in Sect. How to deal with special patients). On the other hand, even in medicine there are no absolute certainties, and triage protocols are left to the judgment of physicians and experts. Here triage tiebreakers such as “narrow social utility, that considers only the differential values of specific social roles and functions and assigns some priority to individuals who fill those roles and discharge those functions” [14] can provide an initial guideline. In the future, we might reach more precise and less controversial judgments (possibly through the implementation of Artificial Intelligence as well).

Some examples can be helpful. Let us suppose that an “important or famous” patient is taken to triage in a hospital where there are no more places available in the ICU and there is no possibility of being transferred to another nearby facility. A situation in which triage kicks in implies that some will certainly not get access to critical care resources.^4^ The problematic situation would arise should the patient not meet the criteria for which they would still be entitled to a ventilator, such as being over 80, having other pathologies or small chances of surviving (a famous young artist, say Aretha Franklin or Pablo Picasso in their forties and in good health, would still be favoured over elderly people in serious conditions regardless of them being a famous artist).^5^

So, what if the patient was Franklin Delano Roosevelt, who suffered from Guillain-Barré syndrome [18] during the Second World War, or Pope John XXIII, who was over 80 during the Second Vatican Council? [19]. And consider someone like Marie Skłodowska Curie, recipient of the Nobel Prize in two different scientific fields [20], or Albert Einstein, a renowned physicist and public intellectual [21], or Andrew Carnegie, a very rich entrepreneur engaged in impressive philanthropy work [22], in the case they did not meet the triage criteria at the moment of admission.

There also may be “intermediate” cases in which the intuition may be less clear-cut and shared. Nobel prize-winning neuroscientist Rita Levi Montalcini or eminent political philosopher John Rawls, over the age of 80, were certainly great figures, but perhaps from the point of view of their contribution to medicine and the humanities they had by then offered the best they were capable of, while remaining important players in the public debate. Actors like Charlie Chaplin would be certainly considered celebrities even at a late age, while former basketball champion Wilt Chamberlain, who had a bad heart condition despite his young age, would perhaps be less likely to be given priority over others in intensive care. But this case can well illustrate how hard it is to make a choice, since it is hard to think of an answer that does not hinge on a subjective (and thus susceptible to bias) judgment of the relative merits of different art forms or fields.

Two considerations can be made here. On the one hand, important or famous people tend to have greater access to care and even emergency treatment when health systems are put under exceptional pressure. This means that major inequalities continue to persist in our society, which are not always justified according to the second principle of Rawls' *Theory of Justice*, whereby any unequal treatment must benefit the least well off (and must be codified in a transparent manner) [23]. In this sense, it would be advisable for everyone to be treated equally even in the context of a pandemic.

Secondly, as we have seen, when there is high pressure on health care systems this often involves choosing how to allocate care instruments that have become scarce in the face of increased demands (as was also the case in the second wave of Covid-19 in the autumn–winter of 2020–2021 and in some countries at the end of 2021).^6^ Now, when famous/important people find themselves in the same 'queue' as ordinary citizens, ethical dilemmas may arise such as the ones we have mentioned above. In general, in emergency situations, whether related to the spread of Covid-19 or during other serious health crises, there may be cases where important or famous people may have no alternative to public facilities that are already full and when they do not fit the criteria for priority of care. In this sense, it seems useful to analyse how such situations might be dealt with from the point of view of medical ethics and the general interest of society.

### An argument for special patients

In a “insufficient ventilators for all” scenario if a special patient is brought to the hospital, what should one do? Faced with such situations, a consistent intuition might be to think that society needs these “important people” for different but clear reasons, and that it would in principle be unacceptable to let them die (in this context, a moral judgement on the individuals as such does not seem relevant, unless they are completely discredited). Most people not directly affected by the virus would want this person to receive priority treatment based on the intuition that certain individuals are not more important per se than other people, but that many people would benefit in many ways from their survival, or more simply many people would like them to survive since their presence or action is appreciated and a source of pleasure.

This can be a widespread feeling—although not shared by all the people depending on the important/famous person involved—and the result of a pragmatic reasoning (a political leader can be pivotal to the ordered functioning of institutions).

What kind of assessment can be made in the face of cases like the ones we have described so far? One possibility, which respects the protocols in place, is to follow the guidelines to the letter and assess whether the “important people” in question can take priority over patients who have already been admitted, strictly based on the clinical parameters established as relevant. But, as said, there is a consistent *prima facie* intuition that people like Roosevelt, Pope John XXIII, Curie, Einstein, and Carnegie should be treated as a matter of priority, not so much for their prognosis or life expectancy, perhaps worse or lower than those of all people hospitalized at the time, but for the contribution to society they could still make, in different forms, albeit for a short time.

Violating the triage protocols—which do not consider social utility as a criterion—and not providing a young patient with access to ICU or taking a respirator away from an elderly patient who would be formally entitled to it in order to prioritize one of the five figures mentioned, however, goes against another *prima facie* strong moral intuition, namely that one should not neglect someone who would be fully entitled to live for the benefit of the community [24].

Guidelines developed so far focus on criteria that affect the individual patient on a scale comparable to that of other potential patients. For example, in conditions of scarce medical resources, priority may be given to those with a higher life expectancy. Alternatively, as seen above, one may employ “clinical suitability”, an assessment involving the type and severity of the disease, the presence of comorbidities, the impairment of other organs, etc. But it has also been proposed that “for patients with similar prognoses, equality should be invoked and operationalized through random allocation, such as a lottery, rather than a first-come, first-served allocation process” [25].

So far, different recommendations have been proposed, also in light of previous analyses [26, 27]. Some scholars have argued for the prioritization of specific categories. “Critical Covid-19 interventions—testing, PPE, ICU beds, ventilators, therapeutics, and vaccines—should go first to front-line health care workers and others who care for ill patients and who keep critical infrastructure operating, particularly workers who face a high risk of infection and whose training makes them difficult to replace” [25].

Other scholars have instead argued against this view. “Priority should not be given to particular groups of patients based on instrumentalist grounds, such as the social value of their jobs. […] As a matter of public law, this type of prioritisation could be challenged for being irrational and/or disproportionate when the evidence is inspected. For example, the evidence might not support the assumption that a doctor who has just recovered from a critical Covid-19 infection will recover the strength to work in time to save additional lives during the pandemic, or that a worker helping to maintain critical infrastructure like electricity services is contributing more to society than a person caring for their friends and family” [3].

One can certainly adopt a strictly welfare-based consequentialist perspective, whereby in emergency situations one must act pursuing the highest overall utility. Some have recently proposed endorsing utilitarianism as “an influential moral theory that states that the right action is the action that is expected to produce the greatest good” and “offers clear operationalizable principles” [28]. The authors have devised an allocation algorithm that can tell clinicians which patients should receive a ventilator in cases of overwhelming demand. The so-called “critical level” utilitarianism considers several rules of thumb related to saving the greatest number of people, probability of recovery, duration of treatment, resources, life expectancy, quality of life, and social benefit. But the authors acknowledge that “developing rules of thumb for assessing social worth is ethically and epistemically complex, liable to abuse and difficult to enforce fairly. Critical level utilitarianism would like not to endorse such priority rules”.

Indeed, there are significant objections to the prospect of a utilitarian/consequentialist calculation that takes non-clinical aspects into account. In fact, to operationalize—so to speak—the value of an individual in terms of the contribution they can give to society, especially when specific areas are involved (think of a philosopher *vs* a former basketball champion) is notoriously difficult and controversial. Some might think that an important philosopher would make a greater, albeit indirect, contribution to people's overall well-being, but the global mourning following the premature loss of retired basketball star Kobe Bryant may offer some indication of the importance that former athletes continue to have in society. This indicates how much emotional aspects may weigh on decisions and how paternalistic it may be to place a higher value on academia than on popular culture. If one is willing to take into account the current popularity of a person, it seems however that social media 'likes' may not be the ultimate criterion for an ethics committee's choice, although social media are really a proxy of a certain kind of popularity.

It would be equally controversial to make a comparison between a self-made person devoted to science or art and someone who inherited a fortune and does not “do” much except give money to charities or universities. Moreover, the “insufficient ventilators for all” scenario in the case of Covid-19 does not leave much time for clinicians to weigh the pros and cons of such a complicated decision. And yet many people are likely to be dissatisfied should “important and/or famous people” be left without care.

At least for some famous patients—for example, President Roosevelt, Pope John XXIII, and Albert Einstein—it seems like that the adoption of a strictly consequentialist criterion can be justified on the basis of the impact and relevance of the role played by these individuals for the benefit of many millions of people. But arbitrariness in determining the importance of an individual may conflict with the prescription of fair and impartial rules. The idea of depriving of emergency care an individual who had legitimately begun to receive them in order to hand over the ventilator to a "famous patient" who would not be entitled to it under the general criteria adopted defies some of our deep-seated moral intuitions, and all ethical perspectives other than pure consequentialism.

It is plausible to think that when faced with an "insufficient critical care resources scenario," if an "important/famous" individual's life is at risk, many people would be willing to donate money or commit some of their time to that individual's survival, as it happened on many occasions. No one (or few) would say that another individual must be let die in order to save the "important/famous" individual in question. To get that individual cured, however, many would be willing to pay a personal price. This may be because of a fondness for a given personality or because of a consideration of its importance to society and culture. This implies that, if all people have equal value and dignity, some make a greater contribution to the wellbeing (variously intended) of others.

Faced with a disagreement between different moral intuitions and between pragmatic needs and the duty to respect fair and universalistic allocation criteria, one can then resort to a supererogatory ethical protocol that allows those who want to pay the maximum personal price in favour of "important/famous" patients. This involves allowing a patient to give up critical care resources in favour of an "important/famous" patient.

The delicacy of this procedure requires not only that it be ethically justified, as we have argued so far, but that it be constructed with the utmost care so that it retains, even in its concrete application, the characteristics of informed and unbiased choice by the patient. In the remaining part of the article, we will therefore focus on the details of this supererogatory protocol, considering extensively all relevant aspects and possible objections.

### How to deal with special patients

The first step is to start from the important/famous patients themselves once recognized as such at hospital triage, by other authorities or institutions, or by their peers, or reported by relatives or carers. In some cases, triage personnel may not recognise an unaccompanied patient as important and/or famous. In that case, if the patient is unable to communicate and nobody reports the case, there is a risk (I)—albeit a low one—that the individual may not benefit from the protocol. On the other hand, there might be a risk (II) that triage personnel over-identify important and/or famous people, by fear of missing out on someone or of being denounced by the potentially important person's family. In general, it seems better to make a type II error—identifying as important a non-important person—than a type I error—namely failing to identify an important person. It is then up to the ad hoc committee (see below) to make a very quick initial selection on the cases that are easy to decide (clearly flagged as unimportant/not famous; flagged as indisputably important/famous) and a more accurate assessment of the other intermediate cases.

The alleged important/famous people can declare: (a) that they accept the general clinical guidelines for the allocation of ventilators and reject any special priority treatment; or (b) that they think that society needs them for their specific skills and abilities or the function they are called upon to perform.^7^

In case (b), a second step is required: does society really need that important/famous patient? Who can make such an assessment? A reasonable hypothesis is to have an *ad-hoc* national ethics committee, composed of a small number of experts in different fields—not only bioethics—with the ability to evaluate the situation effectively and quickly. Because of the delicate role they are called to play, the members of this ethics committee should be, for example, drawn by lot by a triple-numbered panel appointed by the national parliament (for example, 36 for 12 posts). Any issues of bias or inadequate representation should be avoided, making sure that the committee includes male and female members, scholars of different fields and different ages, ethnicity, and sexual orientation as well as exponents of minorities and less advantaged groups.

There is no guarantee that this committee would be made up in an optimal fashion, including all relevant societal domains. However, extensive scrutiny and systemic readjustments would reduce possible bias. The procedure can be further strengthened with an initial list of experts nominated by universities, ethics committees, associations of various kinds and organized interest groups. To this long list, members of Parliament can add a further 20% of names, and the Parliament would then compile the short list of 36 members by qualified majority vote.

As for health professionals in the ICUs, they could be provided with information through briefings, considering that in emergency situations time is short and precious. Health professionals have an important role in the procedure, but they are not the ones who have to make these decisions. Physicians and nurses who disagree with the procedure could invoke some form of conscientious objection (not being forced to disconnect the ventilator from a patient who voluntarily hands it over to an important person) and this should be always allowed. On the other hand, when there is a sudden shortage of critical care resources, physicians today have to make choices for which they have not been given specific instructions. They rely on their training, experience, and conscience. In this sense, one could also preventively involve health staff professional associations (as seen above).

It should not be forgotten that triage protocols stipulate that first the clinical assessment is carried out (according to the criteria mentioned above) and only then, if the situation requires, does one rely on different kinds of tiebreakers. In many hospitals there are dedicated triage teams (whose composition is chosen to avoid conflict of interests between physicians working at the bedside and people they could know—relatives, friends, colleagues). Triage teams make decisions as objectively as possible, considering only clinical aspects. In this sense, the framework proposed here would bypass triage protocols when a potentially important and/or famous patient is identified. At that point, the decision would be referred to the committee set up to assess that kind of patients.

It is worth noting that in 1961, in Seattle, after the invention of the shunt for chronic haemodialysis that could save people otherwise destined to die, a special procedure was implemented to select the few patients that could be admitted to the then scarce and expensive treatment [29–31]. A committee of physicians evaluated candidates by strict medical criteria. People who passed the first screening were further selected by the Seattle Artificial Kidney Patient Selection Committee, composed by a lawyer, a priest, a housewife, a state official, a bank clerk, a trade unionist, and a surgeon. Decisions on whom to admit were based on age, gender, marital status, income, net worth, emotional stability with specific regard to the potential patient’s capacity to endure dialysis, educational background, nature of occupation, and social respectability. The candidates were to provide names of people who could serve as referees.

The protocol was quite different from current triage criteria, which do not consider social and personal features. The procedure adopted in Seattle—where the second group was dubbed “God committee” as it had the power of life and death—was fiercely criticized (although it probably was one of the first, unfortunate attempts to create an ethics committee). In 1972, a law was passed to ensure that all American citizens with end-stage kidney failure could access the new medical treatment thanks to Medicare.

How to allocate scarce dialysis treatment resources in the clinical course of chronic kidney failure entailed a longer time framework compared to acute respiratory difficulties, and one could claim that the same processes would not work equally well in the latter case, let alone the biased evaluations by the “God Committee”. Indeed, until not long ago it would have been impossible to summon a large committee and have them deliberate until a majority opinion is reached quickly enough to make decisions about how to allocate ventilators in a hospital ward. In fact, decisions about ventilator allocation should be made within hours or even, in some rare cases, a few minutes. This concern might be especially salient if the committee consisted of health experts with other critical responsibilities in a situation of healthcare crisis.

But the spread of digital technology that the pandemic itself has accelerated comes to the rescue in such cases. One can assume that the committee would be large enough in number to be divided into two subcommittees, whose members are available for 12 h a day (provided cases requiring their deliberation should still be very rare). Thanks to the digital platforms now available and to smartphones or tablets, a central contact person reached by the hospital in question can arrange the virtual meeting in a matter of minutes, also circulating the patient’s file and some biographical notes about them (for example, an easily accessible Wikipedia page is available for most of the famous people we are discussing in this article). In this way, the committee members could connect and discuss the case very quickly, also depending on the time available in relation to the patient’s diagnosis.

Decisions need to be taken by qualified majority (e.g., two thirds of the members). Utilitarian and non-utilitarian considerations should be involved, and the committee should rely on a procedural process to ensure a quick and reasonable decision is reached. The criteria must include: the objective notoriety of the person and their contribution to their field; the likelihood that this person will continue to make a contribution; their connection with a large group of people; the general impact of their actions (think of a well-known human rights or peace activist, a philanthropist, an artist or an intellectual); and the effect that their sudden death might have (think of a political or religious leader). The criteria in use could also be formalized and rated on a scale of 1 to 5 so as to allow for a quantitative judgement on which to base the decision. In the case of Covid-19, being vaccinated (with the exceptions of those who are not immunized for proven medical reasons) could be a prerequisite.

Once the decision has been made as quickly as possible, depending on the characteristics of the individual case, the record of the committee’s work should remain available to the institution that appointed it (e.g., the national parliament) and the committee should draw up a written report to be made public later. Some privacy issues would remain to be addressed, as the patient giving up the ventilator would not know the identity of the recipient when confirming their previously expressed wishes. Although it is difficult to envisage maintaining complete anonymity in the procedure, the anonymity of both donor and recipient might be the best solution at least for a certain time frame. Full publicity, including the identities of the people involved and the decision of the committee, could later be allowed for reasons of transparency.

The whole process is certainly resource and time consuming, but the establishment of such a committee could also be leveraged as a kind of super-triage at the national level to adjudicate controversial cases of care allocation or urgent medical decisions. The example of the Seattle "God Committee" shows how opening new avenues involves a process of trial and error.

### A supererogatory protocol

If the ethics committee established that society needs the given (important/famous) patient in the “insufficient ventilators for all” situation, a supererogatory protocol could be hypothesized, in which altruistic individuals are willing to renounce their ventilator to hand it over to (important/famous) patients who need it but cannot get it. Notably, altruism is taken to be a special human feature which is widespread and morally appreciated even if it implies (seemingly) irrational acts. Think of a woman running into a burning house to save a child she never saw before. People would praise her, and the more severe the burns, the greater the appreciation for her. If she died, she would be remembered as a heroine [32, 33].

However, it does not seem ethically fair to ask patients who have already been admitted: “Who is willing to give up their ventilator for the good of the community?” when an important/famous patient has passed the above evaluation. This type of procedure would put too much undue pressure on weaker or emotionally unstable people and could also lead to serious abuse on patients. In addition, it would not be easy to practically implement because ICU patients are often sedated and not in their full mental capacity.

One way is instead to establish the possibility, at the time of hospitalization in intensive care, or even before (as it happens for organ donors), to sign a (revocable) commitment, with all the guarantees of informed consent, to give up one’s place to an important/famous patient should there be a lack of respirators. Patients should be given a full explanation of the procedure described above so that any supererogatory waiver is a choice made within a clearly codified framework and is the result of an informed decision. Financial compensation to the family should be prohibited to avoid choices dictated by poverty or pressure from relatives.

The idea of obtaining informed and consensual waivers from patients might raise some concerns about the feasibility of the process. In this sense, some challenges to obtaining proper informed consent in such a situation need to be addressed. Firstly, some severely ill patients could be unable to provide legitimate informed consent at the time of their admission to the hospital. In such cases, it is simply not possible to obtain informed consent of any kind. Such patients cannot express the will to give up their ventilator, unless they have issued a special form of *living will* (more on this below). Furthermore, an individual who has expressed consent to the procedure on admission to hospital and who is not able to confirm their choice at the time of need should not be considered eligible for the procedure, even if their prognosis is poor. As patients on mechanical ventilation are sedated, the potential donor should be aroused, if possible, in order to restate their will and then put under deep sedation if they do so. A patient who renounces their critical care resources would probably die, but it cannot be excluded that they could unexpectedly survive even without a ventilator (or intensive unit care) and then recover.

Secondly, one needs to consider the physical and psychological burden that this process may impose on patients as well as the additional labour that this administrative step would require from hospital staff whose time and energy is already scarce. Is it worthy to require yet another informed consent form process for each Covid-19 hospitalized patient, if the “insufficient ventilators for an important patient” arises, say, in one out of a million hospitalizations? This may be a relevant concern, but there are two possible answers. The additional informed consent part about “important people” should not lengthen the hospitalization process, even though it obviously has to be well explained by the doctors and well understood by the patients. A second solution, which differs from this one and avoids the time concern issue, could be a living will signed by healthy individuals prior to hospital admission.

As said, the assessment of the suitability of the important/famous patients to access this opportunity should be entrusted to a third party (the ad hoc ethics committee). A “Samaritan” patient who has signed the supererogatory commitment to hand over their ventilator to an important/famous patient in an emergency may change their mind at any time, but not according to the identity of the important patient, which should be kept secret from them so that they do not risk being influenced by personal or superficial judgement.

In their living will potential donors of critical care resources should be able to express which categories they would like to exclude from potential beneficiaries, but not from an established list already set. If "politicians" or "businessmen" are, for example, unambiguous definitions, other terms, such as "intellectuals" or "rich" should be interpreted by the committee. Instead, potential donors should not be able to select people or categories to whom they would like to donate their critical care resources, to avoid the undue influence that an "important" person can have on more vulnerable individuals.

It could also be envisaged that on the death of the beneficiary the identity of the former and the donor should be revealed, giving public posthumous credit to the latter (but not before 5 years after the death of the donor, to avoid emotional reactions and minimize legal disputes).

Such a procedure may seem to be geared towards encouraging a voluntary supererogatory practice to which no one can be obliged but which people who are particularly dedicated to the good of society, in full awareness of the risks that this entails, might still want to undertake [28, 34, 35]. In this way, several consistent (coherent) and otherwise necessarily conflicting moral intuitions could find resolution. However, there is no guarantee that there will be people willing to make a supererogatory renunciation, or that they will be there in the places and times when they may be needed.

A way to let people know of the chance of giving over their ventilator without overburdening newly hospitalized patients and health care personnel would be to advertise this possibility with a well-calibrated institutional information campaign, so that whoever wants to do so, can adhere to a specific living will. Such a campaign should be modelled on the informed consent of the scenario presented above but should be filled in beforehand, in the presence of a public official, and should always be revocable. This would avoid the objection raised earlier about the difficulty of collecting informed consent from frail and acutely distressed patients. Those who might agree to give up their ventilator would thus also be more aware of the consequences of such a choice.

An additional criterion could also be introduced to reduce possible prospective misjudgement by healthy people with respect to future decisions. In particular, the living will could contain a requirement that the ventilator (or other medical life support in the case of other diseases) can only be given up if the person is admitted to an ICU with a poor prognosis for recovery (although prognoses in these situations are uncertain).^8^

From a general ethical viewpoint, the question must be asked whether society should allow such supererogatory choices and, if so, whether it should promote them. Indeed, in the absence of a legal framework, in general no one can give up hospital care for the benefit of others, even close relatives. In many countries, people are allowed to renounce healthcare or treatment if they consider them as futile or disproportionate [36–38]. However, it is a different matter to hand over a care instrument to someone else because there may always be a suspicion that the individual is not acting freely and is under some form of pressure or coercion. That is why, in our proposal, the patient’s family should not receive any monetary compensation, nor should the patient know to whom their ventilator is going.

In any case, even if the person signs a specific informed consent or a prior living will, some form of legal framing of the scenario considered here might be advisable, as there might be (rare) legal disputes between the family of the ventilator recipient and the hospital health care staff, or between the patient’s family and the ventilator recipient. If the procedure is properly codified and applied, however, there seems to be no general legal impediment to putting it into practice.

There have already been such cases. In 2020, a 65-year-old nurse in Pittsburgh (USA) claimed to have changed her will to give up her ventilator in favour of a younger patient with Covid-19^9^; in Italy, a 72-year-old Roman Catholic priest apparently turned down his ventilator and died in a hospital.^10^ It is not known whether there have been any judicial enquiries into these cases. Recently, an Italian 91-year-old individual offered to give his vaccine to the mother of a disabled young man who was not yet on the list, but the local health authority did not allow the exchange and ended up vaccinating both at the same time.^11^

Summing up, establishing the possibility of a supererogatory waiver procedure in favour of important/famous patients that cannot receive a ventilator would be a way of encouraging a gesture that many might not even consider, and which would entail important consequences for family members and all those who are close to the “Samaritan” patient in question. Indeed, it could be argued that a just society should not need supererogatory gestures involving a very high risk for those who make them, because such a society would have rules and resources to ensure a fair allocation of rights and opportunities for all its citizens.

Still, emergency situations, such as disaster medicine or the acute phases of the Covid-19 crisis, seem to create painful moral dilemmas to which it is difficult to find a solution that does not involve a decision in favour of some people and to the detriment of others. In this sense, the supererogatory renunciation procedure seems to respect the most widespread moral insights and intuitions, while maximizing the autonomy of any individual, without violating any basic rights in the “insufficient ventilators for all” scenario.

## Conclusion: an open question

In light of the discussion provided, we can still wonder if we really need important/famous patients to the point of violating/changing clinical protocols and guidelines and the equality principle with respect to the right to preserve the fundamental good of life. In an imperfect world, such violations already occur in other areas. The question is whether this is due to a procedural malfunction whereby the ethical standards we should be guided by are violated, or whether it is for moral reasons that justify the unequal treatment.

A purely consequentialist ethics may perhaps argue in favour of replacing patients on the list of access to respirators based on a calculation of expected global utility, but this seems to go against a common deontological restraint, because it is not immediately clear how the sacrifice of one can help many. A good example of this comes from the different responses people give to the main versions of the trolley problem. In the original version of the moral dilemma, most people tend to opt for pulling the lever, redirect the trolley, kill one and save five. In the footbridge version, only a minority of participants are inclined to kill directly the “fat man” so as to save five workers—that, numerically speaking, does not make sense. This is one of the reasons why some scientists have tried to get some help from neuroscience to better understand the way we conceptualize these two very similar scenarios so differently [42].

Experimental data on the trolley problem, indeed, seems to indicate that decisions are influenced both by the implicit meta-ethics adopted by participants and by the specific situation that activates different brain processes. It might therefore be useful to conduct a laboratory simulation of the protocol for famous/important patients in order to understand the intuitive reactions of both health care professionals and the general public to the possibility of giving up a life-saving device in favour of an important/famous person whose identity one ignores.

On the one hand, the formalized possibility of a supererogatory renunciation is in line with *prima facie* consistent moral intuitions. On the other hand, it could also be considered a general incentive to altruistic/virtuous behaviour in general, although this claim is debatable. It is not a shared view that altruism, and especially this kind of radical altruism, should be incentivized. Yet, despite the limitations and risks that have been highlighted, the proposed framework seems to be the preferable course of action in the “insufficient ventilators for all” scenario, if we consider that certain patients are worthy of special treatment.

## Data Availability

Not applicable.

## Abbreviations

ICU: Intensive care units
SIAARTI: Italian Society of Anaesthesia, Analgesia, Resuscitation, and Intensive Care

## Footnotes

1 The issue of shortage is not only linked to the availability of machines but also to the lack of caregivers to operate ventilators and care for the patients. For the sake of brevity, we will use “ventilators” as synonymous with “critical care resources”, a concept which is also appliable to medical emergencies different from Covid-19.

2 See SIAARTI’s recommendations [8].

3 Obviously, from a different point of view, almost all people are “important” to someone and sometimes to a high number of acquaintances. Here, however, we want to focus on people who exert influence on a greater number of other people whom they do not know personally. That said, from a normative point of view, we believe that each person is “important” and worthy of the utmost attention and care.

4 It is appropriate to keep in mind the further ethical challenges brought to the fore by the Covid-19 pandemic, which have been analysed in some recent works; see, among others, [15, 16]. Here we do not discuss the moral difference (if any) between withholding and withdrawing medical treatment, since in our framework it has not a high relevance [17].

5 We are giving examples of historical figures to avoid unpleasant references to living people and not to burden the analysis with emotional elements that could make it less objective.

6 See media reports [15].

7 Some people may declare themselves socially “important” even if they are not well known, such as leaders of associations or religious movements. Another situation that could be considered is one in which a large group of people ask for the importance of a specific patient to be recognized, but we should be aware of the connected risks of entering the realm of popularity instead of importance.

8 Some might wonder whether important and/or famous people, in case they are saved, should commit to making a special contribution in the future. This does not seem to be the case in the framework of supererogatory acts that we propose here. Those who are saved could possibly be warned that if they commit serious crimes or major moral violations—an extreme case of ingratitude to the detriment of society—the commission could reveal the fact that they had benefited from a person's sacrifice when they were ill.

9 See media reports [39].

10 See media reports [40].

11 See media reports [41].
